# Development of a multi-layered psychosocial care system for children in areas of political violence

**DOI:** 10.1186/1752-4458-4-15

**Published:** 2010-06-16

**Authors:** Mark JD Jordans, Wietse A Tol, Ivan H Komproe, Dessy Susanty, Anavarathan Vallipuram, Prudence Ntamatumba, Amin C Lasuba, Joop TVM de Jong

**Affiliations:** 1 Department of Research & Development, HealthNet TPO, Amsterdam, The Netherlands; 2 VU University Medical Center, Vrije Universiteit, Amsterdam, The Netherlands; 3 School of Medicine, Boston University, Boston, USA; 4 Church World Services, Jakarta, Indonesia; 5 Shantiham, Jaffna, Sri Lanka; 6 Burundi Country Office, HealthNet TPO, Bujumbura, Burundi; 7 Sudan Country Office, HealthNet TPO, Yei, Sudan; 8 Faculty of Social and Behavioural Sciences, Utrecht University, Utrecht, The Netherlands

## Abstract

Few psychosocial and mental health care systems have been reported for children affected by political violence in low- and middle income settings and there is a paucity of research-supported recommendations. This paper describes a field tested multi-layered psychosocial care system for children (focus age between 8-14 years), aiming to translate common principles and guidelines into a comprehensive support package. This community-based approach includes different overlapping levels of interventions to address varying needs for support. These levels provide assessment and management of problems that range from the social-pedagogic domain to the psychosocial, the psychological and the psychiatric domains. Specific intervention methodologies and their rationale are described within the context of a four-country program (Burundi, Sri Lanka, Indonesia and Sudan). The paper aims to contribute to bridge the divide in the literature between guidelines, consensus & research and clinical practice in the field of psychosocial and mental health care in low- and middle-income countries.

## Introduction

There is ample literature available to demonstrate the impact of perpetual political violence on child mental health [1,2]. A broad spectrum of consequences have been reported, including disruption of normal developmental pathways [3], breakdown of social structures such as family and school systems [4,5], increased psychopathology such as depression, post traumatic stress disorder and anxiety [6,7], as well as literature stressing the non-pathological nature of children's reactions, such as increased aggression, withdrawal, pre-occupation with negative thoughts [8]. At the same time, there are authors that warn for pathologizing entire populations and advocating children's and community's resilience [9,10]. Patel and colleagues [11] report a vast gap between child and adolescent mental health needs and mental health resources in low- and middle-income countries, advocating for increased promotion and prevention activities. Moreover, there is very little evidence for the effectiveness of interventions in complex emergencies [12]. Concerned about the impact of violence and lack of attention for needed care, the international humanitarian community has developed a framework of protection, that is increasingly incorporating psychosocial and mental health care for children in complex emergencies like war [13-16].

Based on guidelines and research-informed recommendations, the following thematic areas on the provision of mental health and psychosocial support for children in low- and middle income countries (LAMIC) seem to emerge. First, the need for a complementary approach that addresses both individual clinical needs (curative approach) and broader needs of community revitalization (preventative approach) is often advocated [17]. Moreover, interventionists recommend moving from single intervention approaches to multi-sectoral, multi-level, ecological or systems-oriented intervention programs [6,10,18-20], i.e. intervention packages that address multiple types of needs ranging from children at risk to children with psychiatric symptoms with a range of services from broad-access (community-based) to restrictive-access (clinic based). However, besides guidelines and discourse, there are scarce examples of such care systems in practice, especially for children in areas of war [21]. Exceptions are the models presented by de Jong [18] and by Saltzman and colleagues [20], who describe mental health programs in low and middle-income countries (LAMIC).

Second, although there is little uniformity in modality for psychosocial and mental health interventions for children in armed conflict [21], the majority of available guidelines and key publications advocate the importance of; (a) normalization of the child's daily life and recreational activities; (b) social reconnection/reintegration and social support mechanisms; (c) utilization of individual and community coping and resilience mechanisms; (d) discouraging child-family separation because of the important role of caregivers; (e) focus on existing education and health care systems; (f) emphasis on reduction of social discrimination and non-medicalization of problems, and (g) youth participation [1,6,18,22,23].

Third, with criticism on approaches that follow a predominant medical model there has been a growing tendency towards interventions that foster community and individual resilience in LAMIC with limited resources. The resilience paradigm includes a focus on social support systems, community mobilization and strengthening existing coping strategies [23,24]. At the same time, there are numerous publications that warn for an artificial dichotomy and argue that there is a substantial group of children with severe and sustained problems that require more focused care [14,25-27].

Fourth, increasingly, from both humanitarian and scientific literature, there is a call for rigorous evaluation of the effectiveness and efficacy of interventions. Some of the few available evaluation studies for children in LAMIC demonstrate moderate treatment effects [28-31], while some studies show no beneficial effect of treatment [28,32]. A recent systematic literature review into the evidence base of psychosocial and mental health interventions for children in war-affected countries demonstrates that there is a serious lack of rigorous studies. Existing studies evidence mixed results (ranging from no treatment effect to moderate effect sizes at most) and are heavily skewed towards a focus on PTSD symptoms [21].

Fifth, cultural variables play a crucial role in the expression of problems and the relevance and choice of health care options. As a result, assessment and services for affected children need to be adapted to their context, building on local perceptions of needs, traditional notions of healing including reconciliation and cleansing rituals, inter-sectoral collaboration and integration within existing services [18].

This paper describes an intervention model, aiming to translate existing consensus, principles, guidelines and scientific literature into a framework of care provision. The intervention model was implemented in five (post-) conflict settings; Burundi, Sudan, Sri Lanka, Indonesia and Nepal.

## Model presentation

**Figure 1:**
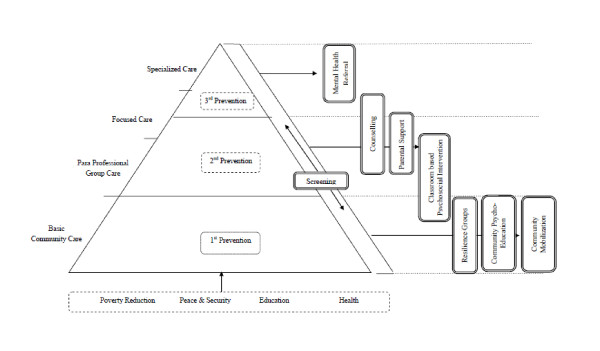
**Comprehensive Child Psychosocial Care**.

To provide mental health and psychosocial support to children in areas affected by political violence we developed a multi-layered care package (See Figure 1; Table 1). A care package approach does not dictate the use of any specific interventions; rather, it prioritizes the facilitated transfer of clients between components along a continuum of care [33]. The first level comprises of interventions targeted to the general population or the whole target group to prevent healthy, albeit at-risk, populations to develop psychosocial problems (e.g. interventions to promote adaptive adjustment and community resilience). The second level consists of interventions that target sub-groups of the population at-risk for developing mental health problems or that demonstrate mild problems (e.g. focused interventions to reduce psychological distress). The third level comprises of interventions that target treatment of sub-groups with severe mental health problems (e.g. specialized interventions to reduce severe psychological distress, suicidal risk and other high-risk behaviors). This package of care was developed before the publication of the IASC guidelines on mental health and psychosocial support in emergencies, but in essence is conform to its principles [14].

**Table 1:** Overview of interventions

Public mental health model	Primary objective	Module	Specific objective	Intervention modality	Personnel	Level of training (indicative)
Tier 1: Primary prevention	Strengthening Community Resilience	Youth groups (Resilience Groups)	• Reduce stigmatization	Group activities		
			• Secondary screening			
			• Increasing social support			
			• Strengthening of resilience • Normalization			
				
		Community awareness raising	• Provide information on CTP	Group psycho-sessions with:	Community	2 weeks
			• Raise awareness on general psychosocial issues	-Teachers	Psychosocial Workers	
			• Raise awareness on community and/or target population- specific topics	- Parents		
			• Mobilization of existing resources and roles	- Community groups		
				
		Community Mobilization	• Utilization of existing community resources	Case-management		

Tier 2: Secondary prevention	Care for children at risk for developing more severe problems	CBI	• Reduce psychosocial distress to sub-threshold	Classroom-based group sessions	CBI Facilitators	10-12 days (with subsequent regular 4-day booster courses)
			• Reduce risk of mal-adaptation • Facilitate resilience and normalcy			
		
		Parent/Family Intervention	• Support child-parent relationship • Child rearing support	Home visits or family sessions	Counsellors	4-6 months
		
Tier 3: Tertiary prevention	Advanced care for children with severe distress	Counselling	• Care for children with more severe problems	Individual or group counselling Case-management		
		Referral to external services	• Specialized care (formal and informal) for severe problems			

Tiers 1, 2 and 3	Improving access to, and quality of, care system	Monitoring and Evaluation	• Determine reached population	Questionnaires	Service providers and beneficiaries	n.a.
			• Evaluation of services			
			• Provide overview of results			
		
		Screening	• Detection of indication for treatment	Child Psychosocial Distress Screener	CBI facilitators/Community psychosocial workers	2 days
		
		Clinical Supervision	• Continued learning • Clinical support through case discussions	Group inter-vision meetings	Mental health professional (incl. experienced counsellors)	Significant clinical experience
			• Support to service providers			
			• Project implementation issues			

*Note*. Counselling is here presented as a tertiary intervention, but as is represented in figure 1, it can also serve as a secondary prevention intervention.

In this paper we use the composite term 'mental health and psychosocial support', to indicate overlapping concepts that refer to a broad concept that encompasses 'any type of local or outside support that aims to protect or promote psychosocial wellbeing and/or prevent or treat mental disorders'. In turn, 'psychosocial' is defined as the close relation between psychological factors (emotion, behavior, cognition) and the socio-cultural context [14,34].

The care package aimed to: (a) increase community awareness on children's psychosocial and mental health problems; (b) mobilize coping strategies and community resources; (c) increase social support systems, and (d) reduce psychosocial distress and severe psychological difficulties amongst children. In doing so the multiple interventions were structured within an interconnected and complementary care system working on different interdependent ecological levels. All services were provided by trained teams of local professionals. Below we will present the content and rationale for the different components of the package (See http://www.psychosocialcarechildren.org for detailed description).

### Community sensitization and psycho-education (tier 1)

The mental health needs of children are seriously underserved mainly due to limited services and resources. But even if available, access to care is minimal. Review of the literature on public awareness of child and adolescent mental health presents the following as the main barriers to care [35]: (1) lack of awareness by parents and children of mental health disorder and services and, (2) lack of understanding by primary health care workers and teachers of mental health problems; (3) the stigma and discrimination related to mental health [36]. Increased public knowledge has a significant effect on help seeking.

Within the care package, the combined sensitization activities aimed to achieve the following. First, they aimed to promote project acceptance. Political, cultural and ethical acceptability of proposed services had to be addressed before starting actual service provision [18]. Second, extensive explanation of mental health and psychosocial care and problems (and its origins) in order to reduce stigma attached to these concepts and care. For example in Sudan and Burundi providing information about the nature and treatment of epilepsy was essential in order to provide appropriate services. Also, misconceptions about mental health problems among community members prevented for example the reintegration of ex-child-combatants in Nepal. Third, increasing understanding and identification of psychosocial issues and problems of children, in order to increase normalization, acceptance and (if indicated) referral of such complaints. Fourth, to raise awareness on psychosocial issues specific to certain communities or target groups. Psycho-education on issues such as alcoholism, child rearing or conflict mediation, related to the psychosocial wellbeing of the community at large, in turn were expected to have an impact on the well being of children. Fifth, it aimed to mobilize existing community resources and roles. Discussion with relevant stakeholders aimed to mobilize existing coping and healing strategies to support children with problems and vulnerable families.

*In practice*: A radio program in Sudan on psychosocial problems of children, as well as the care program, was broadcasted weekly. Media proved a strong tool to heighten public awareness by reaching large numbers of people and it helped in mainstreaming the new intervention program within a volatile setting.

### Screening (tiers 1 and 2)

When establishing a multi-layered care system a screening mechanism can facilitate the process of care allocation, especially when targeting large at-risk populations, as is the case in post conflict situations. The use of validated screening tools has been advocated as it permits early detection of at-risk populations and subsequent treatment planning [1,37,38]. Especially when a lack of mental health professionals prevents clinical assessments within large populations [36] and lay-screening is largely absent due to un-awareness or stigmatization [20]. At the same time a screening procedure should only be employed if certain criteria are adhered to [39], which include, among others, that the screening is only done if appropriate treatment is available upon screening, that the screening instrument is safe, simple, acceptable and has demonstrated validity and that cost of screening should be balanced in relation with expenditure of treatment. The screening instrument and procedure that we employed meets most of these key criteria.

The Child Psychosocial Distress Screener (CPDS) is a multi-indicator and multi-informant instrument that measures non-specific psychosocial distress [38]. It is developed in and for non-Western complex emergency settings, and is contextualized through the inclusion of setting-specific probes. The CPDS is a brief community screener that includes the child's appraised traumatic and current distress, resilience components like coping and social support and school functioning. Validation studies have demonstrated that the CPDS has sound psychometric properties with good accuracy for detecting indication for psychosocial care, and robustly measures a common core theoretical construct across settings, albeit with context specific manifestations [38,40].

Within the care package screening followed several steps. First, pre-screening briefing was conducted for the children, teachers and the parents to explain the reasons and procedure of screening to minimize false expectations and socially desirable answering and increase acceptability. Second, primary screening was conducted by administering the CPDS and assessment of exclusion criteria for group intervention. These included; (a) the inability to function in a group setting (e.g. violent behavior), and (b) a group of psychiatric problems (mutism, mental retardation, substance abuse, epilepsy without medication, panic/phobic disorders, and child psychosis) which were expected to obstruct participation and benefit from a group intervention. The CPDS score (following a locally validated cut-off score) and exclusion criteria gave an indication for; non-curative group activities; group-based psychosocial intervention; counseling or referral (see below). Third, during each of the consequent interventions, service providers assessed the child with the aim to determine whether a more (or less) specialized, or other, intervention was indicated.

*In practice: *Screening data from the four countries demonstrated throughout the program (*n *= 29,292) demonstrated that approximately 40% of children were screened positive (Burundi 41.1%; Indonesia 42.4%; Sudan 38.1%; Sri Lanka 42.4%) [41].

### Community Mobilization: Working with existing resources (tier 1)

It is obvious that communities should not be considered devoid of resources in dealing with psychosocial and mental health problems. The available resources, or ecological resilience, can be defined as those assets and processes existent on all social-ecological levels that have shown to have a relationship with good developmental outcomes after exposure to situations of armed conflict [24]. Ecological resilience represents a reservoir of factors at different social-ecological levels that can enhance psychosocial wellbeing. Children under strain can seek out and utilize resources from this reservoir to enhance their chances of retaining or obtaining psychosocial wellbeing. From a primary prevention perspective there are several reasons to focus on strengthening ecological resilience. The impact of war on social structures has often disrupted the functioning of exactly these existing resources. Moreover, it encourages integrated, non-vertical care systems, which are likely more sustainable and cost-effective. Working with traditional healing and religious practices, availing norms and coping is preferred for reasons of availability, sustainability and cultural sensitivity [23]. Fourth, active community involvement taps into the responsibility of the community to support, reducing dependability on external service/resources.

Within the care package different strategies have been employed to strengthen ecological resilience and community self-help strategies, such as; (1) assessment of existing healing practices and community services; (2) creation of resource maps and subsequent development of a case management system; (3) negotiation and involvement of community stakeholders; and (4) collaboration and referral to existing care and (traditional) healing services. Examples included the organization of inter-school drumming, dancing and football contests in Burundi; tapping into religious activities in Indonesia and linkage with mother groups and using extended family structures in Sudan.

*In practice: *In Burundi 'child-to-child' networks were established. These peer groups organized themselves to identify children and families within their communities in need of support and subsequently to arrange or advocate for assistance (e.g. gathering fire wood, harvesting, fetching water). The peer groups were also involved in arranging sport events, cultural activities, and recreational activities at schools.

### Child Resilience Groups (tier 1)

Resilience can be defined as "good outcomes in spite of serious threats to adaptation or development" [42]. Increasingly within mental health and psychosocial support programs for children in LAMIC settings, attention is shifting from focus on treatment of symptoms to promoting resilience [6,43]. Positive peer relations and group activities have been identified as protective factor for children in adversity, contributing to restoring damaged social fabric by developing stronger trusting relationships [10,24].

Within the care package Child Resilience Groups was the generic term for a set of semi-structured group activities for those children without indication for focused mental health and psychosocial support. The first aim of these group activities was to strengthen existing resilience by encouraging social support systems, engagement in recreational or traditional activities and normalization through peer-group discussion and activities. The second aim was to reduce stigmatization due to (non-) enrolment in psychosocial interventions for both the indicated and non-indicated groups of children. A potential risk of focused mental health and psychosocial support was that the indicated group was stigmatized or that the non-indicated group was 'envious' for not receiving any care. Two strategies to overcome such challenges included community sensitization (see above) and ensuring that all children received some intervention, matched for the level of need for psychosocial care. Third, structured and recurring non-therapeutic group activities provided the opportunity for case identification. Initial screening in the care package was done through the brief procedure described above, which inadvertedly resulted in a group of 'false positives' and 'false negatives'. Within the context of non-therapeutic groups, facilitators could still identify, through behavior observation, the false negatives and subsequently refer to more active-therapeutic care. Practically, implementation happened through group formation based on the screening outcomes and participatory determination of activities, which ranges from recreational and traditional activities to theme-based discussion groups (e.g. life-skills focused discussions combined with songs and dances in Sri Lanka; drumming and dancing or football-practices resulting in inter-community competition or presentations in Burundi).

*In practice: *While content was variable across the countries to allow for group- or community-specific activities, a common structure was followed in that groups would convene once per week for the 5 weeks (to equal the duration of the classroom-based intervention thereby minimizing the possible stigmatization of either group). Beneficiary perspectives across the settings (n = 24,690 throughout the program) displayed high levels of participant satisfaction (95% Burundi, 97% Indonesia; 95% Sudan; 86% Sri Lanka).

### Classroom Based Intervention (CBI) (tier 2)

Secondary prevention interventions, in conflict-affected settings with limited resources, are typically large-scale low-intensity interventions. Consequently, it concerns interventions that can be carried out by para-professionals and within a community setting. Schools are often recommended as the setting of choice for psychosocial support interventions as it offers a familiar, non-stigmatizing setting and provide the broadest access to children and their families [20,44,45]. Moreover, usually group work rather than individual work is preferred; because (a) group members can recognize that they are not alone with their problems, (b) group members can learn new strategies and coping skills from each other, (c) the group can function as a place to try out new problem-solving skills, and (d) economic constraints and limited available mental health professionals [1].

CBI is a 15-session classroom or community-based intervention, involving a series of highly structured expressive behavioral activities, which aims at increasing children's capacity to deal with the psychosocial problems that having been/being exposed to extreme stressors can cause [45]. CBI's objectives are to; (1) reduce the risk of mal-adaptation; (2) facilitate resiliency & return to normalcy; (3) facilitate empowerment and mastery; (4) use a natural learning environment, and; (5) screen for high risk youth. It includes mainly group activities such as cooperative games, music, drawings and psychodrama that focus on stabilization and safety, individual coping strategies, traumatic exposure narratives, and future-oriented resources. CBI implementation included the following subsequent steps; (a) initial target area selection based on public health criteria [18]; (b) obtaining permission for care provision from local authorities; (c), review and adaptation of intervention within the give context; (d) skill-based capacity building of the facilitators; (e) coordination with school principals, teachers and parents for practical arrangements; (f) pre-intervention community awareness raising (see above); (g) 1-2 hours sessions, spread out over 5 weeks, within the school premises; (h) post-intervention follow-up and referral when indicated and finally structural monitoring and evaluation.

*In practice: *Two cluster randomized controlled trials studied the efficacy of CBI as part of this program [30,31]. Results show that CBI is moderately effective in reducing PTSD and maintaining hope in Indonesia, while reducing psychological difficulties and aggression among boys and increasing pro-social behavior among girls in Nepal.

### Parental support (tier 2)

Targeting families, and specifically parents, to improve the psychosocial wellbeing of children is recommended for several reasons. Primarily because parents, as the natural child raisers, are influential mediators of children's reactions to (non-familial) violence [46]. The family's stability, safety, parental wellbeing and emotional sensitivity are crucial predictors of social-emotional adjustment. Moreover, the family system has often been put under enormous stress as a result of war and may need support in undertaking this role. At the same time, adults may underestimate, deny or be unaware of the difficulties their children are experiencing or vicariously contribute to the children's problems. Dybdahl [29] further notes that parental capacities, including healthy parent-child interaction, are affected when parents are suffering from surrounding violence. Wallen & Rubin [46] have summarized the role of the family in mediating negative effects of violence as follows: (1) physical availability of the parents; (2) protection and physical safety by parental awareness about potential dangers and subsequently install rules, education and supervision; (3) support in working through traumatic events through communication and emotional sensitivity; (4) child rearing that fosters moral development to counter-balance the moral erosion as a result of conflict; (5) models of positive coping regarding safety, emotion regulation and sense of control.

In the care package the provision of family-oriented supportive counseling, mostly through home-visits, focused on parental capacities. Family support was integrated in the counseling process as a treatment strategy for children who were referred in need of more focused care. The decision to include the family was made by the counselor upon assessment of the problem and developing a subsequent plan of action. At a minimum family support meant psycho-education sessions with parents to increase problem identification and awareness, and subsequently extending to child rearing support (i.e. simple behavior modification techniques), linking the family to existing services and social support systems, and provision of family problem solving support (based on existing parental coping strategies). Additionally, individual counseling for parents was offered, aiming to increase their wellbeing, in turn increasing their capacity to engage in their caretaking roles. There was no systematic way of providing family support to all children who presented with more severe problems, for reasons of feasibility. Future efforts should aim to address this gap, especially given recent research that associates family inclusion in the counseling process with more positive client outcome trajectories [47] and the emerging evidence for the effectiveness of parenting training interventions [48].

*In practice*: A flipchart with printed drawings was developed to be used as a pictorial psycho-education tool for parents in Nepal. The tool 'the role of parents in changing children's behaviors and feelings' aimed to facilitate discussion with parents around issues of wellbeing of children and child rearing.

### Psychosocial counselling (tier 2 and 3)

Moving up the public mental health pyramid, interventions get increasingly focused to treat more severe problems. Counseling is a relatively easy-access level of care that targets more severe forms of distress, both non-specific and common mental disorders, forming a link between informal and formal specialized care structures. In the humanitarian field the term counseling is often so widely used, that it is depleted of meaning. In the literature it is often associated with 'trauma-counseling' [9,49]. We use counseling as a non-specialized individual (or group) supportive and problem-management intervention. The core practice elements are structured problem-solving, symptom management, psycho-education, and emotional support in a relationship offering trust and hope through applying a set of non-specific therapeutic skills, intercultural sensitivity and structured steps that aim to reduce both stressor-induced symptoms of distress as well as, whenever possible, problem situations [50,51]. For application within a non-Western setting, basic concepts from medical anthropology, such as working with clients' illness experiences, explanatory models and idioms of distress, have been included [cf. [52]].

Within the care package, we followed a 4- to 6-months skill-based capacity building approach, which has been described elsewhere [53,54], to train a core group of para-professional counselors. Children who appeared severely distressed at the time of screening or for whom group interventions are contra-indicated, who displayed severe problems during other interventions, or who demonstrated no improvement as a result of the group-based psychosocial care (i.e. follow-up) were indicated for counseling. Practically, children were referred as a result of community psycho-education or the structured groups activities (CBI, child resilience groups etc). Counselors, through a series of sessions, would subsequently develop treatment and case-management plans with specific intervention strategies based on the type and severity of the problems. A research involving eleven *n *= 1 studies aimed to create better understanding of the treatment processes of counseling in LAMIC has resulted in a set of key treatment ingredients that appear associated with client change patterns, i.e. client centeredness, therapeutic alliance, problem solving, and trauma-focused exposure [47].

*In practice: *In Nepal, a process of cultural adaptations has yielded changes in intervention strategies including a shift of focus from intra-psychic or cognitive processes to concrete problem solving, application of specific counselling techniques (e.g. relaxation exercises, yoga exercises), and the inclusion of a thorough psycho-education component [55].

### Supervision

Supervision was an overarching component within the psychosocial care system, to ensure continued development of knowledge and competence, and professional clinical support for the service providers, mainly through case discussions. It provided a forum for the supervisee to raise their concerns. Specifically, supervision was conducted to; (1) enhance and ensure the quality of work, in line with ethical standards, by review of counselling and support skills and processes; (2) enhance the professional and personal capacities and increasing the self-awareness of the service provider; (3) help to better understand and deal with the problems of their clients; (4) monitor and evaluate service provision; (5) provide emotional support, through encouragement and empathetic understanding, to the service providers themselves. Van der Veer and colleagues [56] provide a good overview of provision of clinical supervision in areas of armed conflict. Additionally, especially in non-Western settings, implementation of community-based services raises many organisational and operational issues, which were addressed through supervision meetings. In practice supervision consisted of (bi-) weekly meetings with the service providers and regular visits to the field by the supervisors.

### Clinical care (tier 3)

Increasingly specialized clinical psychological or psychiatric assistance were required when the needs exceed the capacity of existing primary and secondary level services. While this level of care is indicated for a relatively small percentage of the affected population, it may still concern thousands of individuals in most large emergencies [14]. Due to the limited resources n LAMIC this is the level of care that is most difficult to provide. At the same time it is also the level of care much needed to reduce high levels of burden of disease that child mental health problems present to society [57].

Within the care package, children with severe mental health problems were identified during the screening procedure or during the course of the offered interventions. Due to a scarcity of skilled mental health professionals and the inability to raise such capacity on the short term, tertiary service provision was limited and dependent on existing formal mental health care systems in the respective countries. Two strategies were used for this high-risk group; utilization of a professional network of mental health specialists, and if unavailable internal referral to the program's most senior/experienced counsellors. Collaboration with hospital-based multidisciplinary teams of professionals in Sri Lanka were an example of the former.

### Implementation

**Figure 2:**
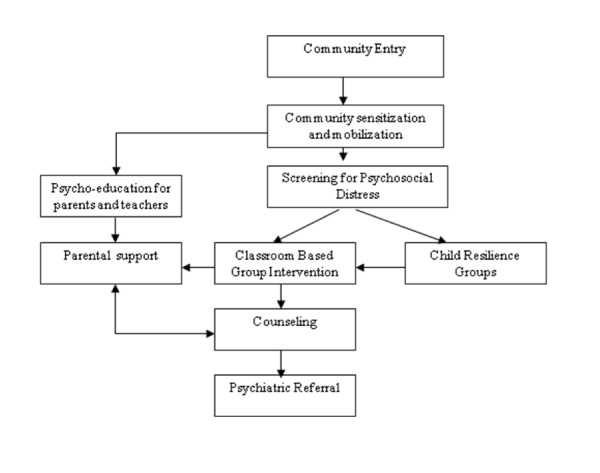
**Overview of implementation**.

Planning and implementing a multi-layered mental health and psychosocial support system depends to a large extent on the specific context, needs and resources. At the same time, in practice, a common framework for developing or implementing such system is advantageous. This section gives an overview of the generic modality of implementation (see Figure 2). Initial preparatory work included need assessments and social mapping, recruitment and structured two-levelled capacity building of service providers. Subsequently, proposed services were presented to local authorities (i.e. education and health) for permission and collaboration. In each of the countries, both the school-based and other interventions needed support and involvement of local government structures. With mental health services still carrying risk of stigmatization, screening and clinical services in any new community was preceded by community awareness raising about psychosocial issues, screening and planned interventions. This was essential in avoiding misconceptions about the interventions. Upon pre-screening briefing of parents, teachers and children, groups of children were selected to undergo the brief screening procedure to allocate services, specifically, the Classroom Based Intervention; child resilience groups, counselling or referral to existing resources or specialized mental health care. While services were ongoing, community psycho-education was provided to parents and other community members to promote the role of parents and existing community resources in supporting children. [See Additional file 1]. We used schools as the entry point to the community-based care system to emphasize children's natural environment and promote normalcy. Parallel and ongoing attention was given to issues of quality control, including continued capacity building of service providers, clinical supervision, structured monitoring and evaluation, and efficacy research [30,31].

## Discussion

In this paper we have argued for a multi-layered mental health and psychosocial support system for war-affected children. Specifically, we have adopted a public health model, aiming to maximize the number of children reached with the limited resources available. This has resulted in a three-tiered system of interventions with different intervention or therapeutic foci; (a) community-based interventions to strengthen resilience; (b) group based interventions to reduce moderate level psychosocial distress, and; (c) focused interventions to address severe distress and high-risk populations. A strength of the approach is that it aims to combine often diverging or unconnected approaches; combining vulnerability and resilience perspectives, targeting of current life stresses as well as exposure to traumatic events, and a focus on new interventions alongside existing resources in the community. Moreover, it provides a replicable working model for multi-layered care in LAMIC settings.

Outcomes, evidence for effectiveness of interventions as well as adaptations of this approach are currently being assessed and are presented elsewhere. Nonetheless, several challenges to this approach can be noted. First, using schools as the entry point for service provision risks overseeing non-school going children. For example in Indonesia, qualitative research showed that a specific vulnerable group concerned children who dropped out of school [58]. Second, a care package approach, even with non-specialized paraprofessionals, may be difficult to sustain with limited financial resources. Cost analyses will need to inform about notions of feasibility in resource poor settings. Third, a common model risks being incongruent with the principle of cultural sensitivity. Careful attention should therefore be given to utilizing such model as a framework within which interventions and implementation is contextualized, based on existing needs and resources. Fourth, sustainability of a system of care will depend in part on the level of integration with existing systems of care. A stand-alone care package risks fragmentation and competing parallel care systems solely dependent on outside financial and technical inputs. Moreover, integration of a care package into existing community and government systems tends to reach more people, be more sustainable and carry fewer stigmas [14]. Although much effort was undertaken to integrate the project in existing community-based systems of care, more efforts need to be undertaken to integrate the above described care system in governmental systems of care and policy. Fifth, and related, the here-described model lacks to specify further linkages with other sectors, i.e. livelihood or peace-building programs (see also Figure 1), considered particularly important in settings of extreme poverty. For example, in line with de Jong [18], Wessells [16] argues for integrated and inter-sectoral collaboration, in which livelihood or infrastructural programs complement psychosocial support (as well as vice versa) in that they often address pertinent distress within poverty-stricken populations.

The above points demonstrate that the presented model is by no means a finalized product; rather it is a framework that in future years needs to be developed and adapted further, at each of the prevention levels. In light of these limitations it is important to note that the paper aims to present an example model that needs to be further developed, adapted and researched. At the same time it aims to demonstrate that carrying out a multi-layered care package is a feasible alternative to a single intervention approach.

In summary, given the gross lack of mental health infrastructure and human resources a core question is how to organize and deliver psychosocial and mental health services for children in conflict affected settings. It is not sufficient to demonstrate that an isolated intervention is effective in reducing a specific disorder among a given sub-population. Above all, we need to demonstrate convincingly that we have a system of preventive and curative interventions that not only address a range of needs but also attend to the mechanisms of care delivery. This paper has described an effort to develop a replicable care package for children in complex emergencies, presenting a framework on how to deliver and organize psychosocial and mental health care. It has employed a care system approach which facilitates transfer of beneficiaries between components along a continuum of multi-layered care, combining preventative and curative interventions, with different care components targeting different sub-populations.

## Declaration of competing interests

The author(s) declare that they have no competing interests. The funding agency had no role in the decision to submit the manuscript for publication, nor any role in writing the manuscript.

## Authors' contributions

MJ, WT, IK, DS, AV, PN, AL, JdJ have made substantive contributions to the conception of the presented model; MJ, WT, IK, JdJ have been involved in drafting the manuscript or revising it critically for important intellectual content. All authors read and approved the final manuscript.

## Supplementary Material

Additional file 1 **Program flowchart**. The figure provided presents a flowchart of the different components of the care package. Click here for file
