# Supplementary figures and images for: Development of a multi-layered psychosocial care system for children in areas of political violence
Source: Int J Ment Health Syst. 2010 Jun 16;4:15. doi: 10.1186/1752-4458-4-15 (PMC2907307; doi:10.1186/1752-4458-4-15)

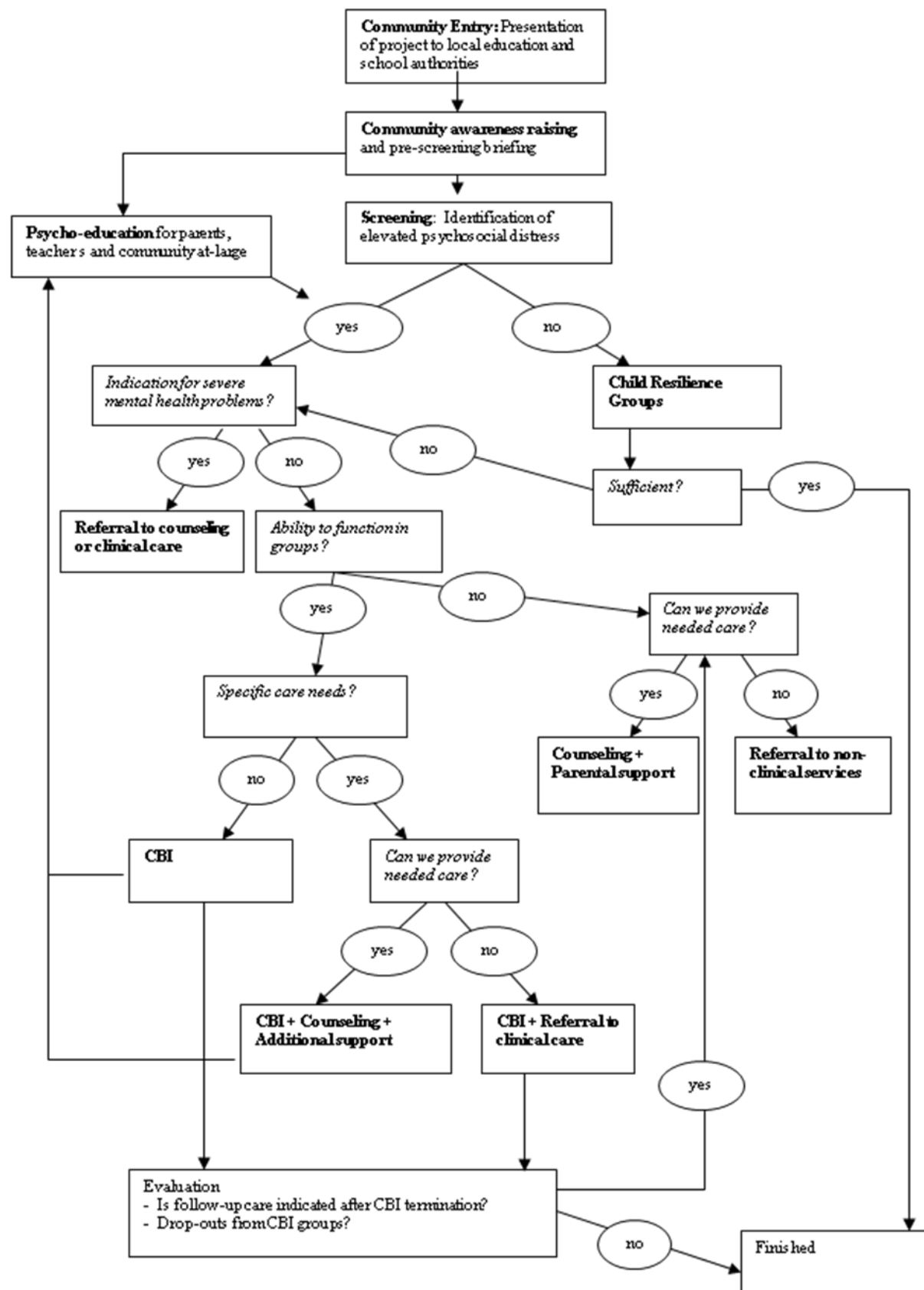

Supplement: Additional file 1 — Program flowchart. The figure provided presents a flowchart of the different components of the care package. [file 1752-4458-4-15-S1.PDF]
